# Minority health social vulnerability index and long COVID illness among a statewide, population-based study of adults with polymerase chain reaction-confirmed SARS-CoV-2

**DOI:** 10.1186/s13690-025-01553-z

**Published:** 2025-03-10

**Authors:** Soomin Ryu, Kristi L. Allgood, Yanmei Xie, Robert C. Orellana, Nancy L. Fleischer

**Affiliations:** 1 Department of Epidemiology, School of Public Health, University of Michigan, Ann Arbor, MI USA; 2 Department of Epidemiology and Biostatistics, School of Public Health, Texas A&M University, College Station, TX USA; 3 CDC Foundation, Atlanta, GA USA; 4 Michigan Department of Health and Human Services, Lansing, MI USA

## Abstract

**Background:**

The COVID-19 pandemic has disproportionately affected socially vulnerable communities. Some individuals experience persistent symptoms and conditions of COVID-19 illness known as long COVID. As little research has examined how social vulnerability is related to long COVID, we studied this topic using Minority Health Social Vulnerability Index (MHSVI), specifically created for the COVID-19 pandemic in the U.S.

**Methods:**

We merged county-level MHSVI data with population-based data of Michigan adults with PCR-confirmed SARS-CoV-2 infection between March 2020 and May 2022 based on respondents’ county of residence. We examined the relationship between county-level MHSVI (binary: high social vulnerability ≥ 75th percentile) and two long COVID measurements, assessed a median of 18.8 months after their initial infection: (1) ongoing long COVID (yes/no) and (2) long COVID diagnosis (yes/no). We conducted modified Poisson regression models with robust standard errors to estimate prevalence ratio (PR) between associations of MHSVI and long COVID overall and by six MHSVI themes (socioeconomic status, household composition/disability, minority/language, housing type/transportation, healthcare access, medical vulnerability), adjusting for individual-level and county-level covariates.

**Results:**

Living in high MHSVI counties was not associated with ongoing long COVID or long COVID diagnosis. However, the associations differed by theme of MHSVI: respondents in highly socially vulnerable counties assessed by medical vulnerability had 1.32 times higher prevalence of long COVID diagnosis (95% CI:1.12 − 1.57). There were no statistically significant associations in other themes after the adjustment for covariates.

**Conclusions:**

Our findings suggest the importance of upstream social determinants of health during public health emergencies and provide evidence that medically vulnerable communities need additional public health resources to cope with long COVID among their residents.

**Supplementary Information:**

The online version contains supplementary material available at 10.1186/s13690-025-01553-z.

**Table:** 

Text box 1. Contributions to the literature
• There is limited public health research on how area-level social vulnerability is related to long COVID.
• This study used population-based data of Michigan adults with PCR-confirmed COVID-19 to find that living in medically vulnerable areas was associated with long COVID diagnosis.
• Public health authorities should provide medically vulnerable communities with more resources and support.

## Introduction

The COVID-19 pandemic has disproportionately affected communities that are economically and socially vulnerable [1, 2]. At the beginning of the pandemic, there were wide disparities in access to COVID-19 testing by geographic area, particularly in racial and ethnic minoritized communities, rural areas, and areas with low-income residents [3, 4]. Moreover, vulnerable communities with higher levels of poverty, household crowding, racial and ethnic minoritized populations, and economic segregation were more likely to have higher rates of COVID-19 cases, hospitalizations, and deaths compared to better resourced communities throughout the pandemic [3, 5–8]. The disproportionate burden of the COVID-19 pandemic among socially vulnerable communities has been explained in part by structural inequities, such as access to high-quality education and employment, safe working and neighborhood environments, transportation infrastructure, health insurance, and healthcare facilities [9].

The disproportionate burden has also been observed for post-acute sequelae of SARS-CoV-2 infection (PASC), which is also known as “long COVID”. Long COVID is a post-viral condition, defined as experiencing persistent signs, symptoms, and conditions of COVID-19 illness after the onset of an acute SARS CoV-2 infection [10]. Approximately, 15% of U.S. adults reported they have ever experienced long COVID according to the U.S. Household Pulse Survey (2022–2023) [11]. Long COVID symptoms may affect multiple organ systems [10] and limit an individual’s daily activity, psychophysical and occupational performance, and social roles [12–15]. Previous studies have highlighted that older age, being female, having lower educational attainment, living in rural areas, experiencing severe acute COVID-19 illness, having pre-existing health conditions, and lack of vaccination are risk factors for long COVID [16–21]. As these individual-level risk factors for developing long COVID are closely related to structural inequalities that could be measured at the area level [9, 16–21], it is necessary to study the relationship between area-level social vulnerability and long COVID.

Several studies have demonstrated that social vulnerability is related to COVID-19-related health outcomes using area-level measures such as the Area Deprivation Index or the Social Vulnerability Index (SVI). Communities with higher social vulnerability are more likely to have higher COVID-19 incidence [5, 9, 22], hospitalization for COVID-19 [23], new mobility disability after COVID-19 diagnosis [24], COVID-19 mortality rates [2, 8, 25], and COVID-19 vaccination hesitancy [26]. However, surprisingly little research has examined how social vulnerability is related to long COVID [27]. Individuals living in socially vulnerable communities, where COVID-19 incidence rates are higher [5, 9, 22] vaccination rates are lower [26] and high-quality healthcare resources are limited [9], may be more likely to experience long COVID compared to socially advantaged communities.

The current study examined the association between county-level social vulnerability and individual-level long COVID by merging data on the Minority Health Social Vulnerability Index (MHSVI) and with population-based data of adults with polymerase chain reaction (PCR)-confirmed SARS-CoV-2 infection between March 2020 and May 2022 in Michigan. We also studied the relationship between social vulnerability and long COVID by each of the six themes of MHSVI: (1) socioeconomic status, (2) household composition and disability status, (3) racial and ethnic minority status and language, (4) housing type and transportation, (5) healthcare infrastructure and access, and (6) medical vulnerability [24, 28]. We hypothesized that individuals residing in counties with a high level of MHSVI would have a higher prevalence of long COVID compared to individuals residing in counties with a low-to-moderate level of MHSVI. Our work extends existing knowledge about the social determinants of health and health equity and provides evidence about the relationship between social vulnerabilities and long COVID.

## Methods

### Individual-level data

 Michigan COVID-19 Recovery Surveillance Study (MI CReSS) is a statewide representative survey of adults 18 years and older with a PCR-confirmed SARS-CoV-2 test in the Michigan Disease Surveillance System (MDSS). A stratified probability sample of eligible adults was selected from 13 geographic strata, including six public health emergency preparedness regions (1, 3, 5, 6, 7, and 8) [29], six counties in southeast Michigan (Macomb, Oakland, Saint Clair, Monroe, Washtenaw, and Wayne [except Detroit]), and one city (Detroit). Sixteen sequential cross-sectional samples were drawn over time with a base number of 50 − 70 individuals from each geographic region, while the remainders of the sample were drawn proportionally based on overall case counts within each area.

Non-institutionalized adults were eligible for the sampling frame if they: (1) had a PCR-confirmed SARS-CoV-2 infection between March 2020 and May 2022, (2) were alive at the time of baseline survey, and (3) had a valid phone number and zip code or county information in the MDSS. Respondents completed the questionnaire either (1) online in English or (2) over the phone with an interviewer in English, Spanish, or Arabic. We did not include adults with SARS-CoV-2 infection based on at-home antigen tests. Respondents completed baseline surveys between June 2020 and December 2022, and follow-up surveys between January 2022 and November 2023. The median time from COVID-19 illness onset to baseline survey was 4.4 months (IQR = 3.4–5.7 months) and the median time from COVID-19 illness onset to follow-up survey was 18.4 months (IQR = 14.9–21.4 months). A total of 5,521 adults completed the baseline survey for a response rate of 32.1%, and 4,100 adults completed the follow-up survey for a response rate of 80.5% (American Association for Public Opinion Research response rate #6) [30]. All respondents provided consent to participate. The University of Michigan Institutional Review Board deemed this study exempt due to the use of secondary de-identified data.

**Fig. 1:**
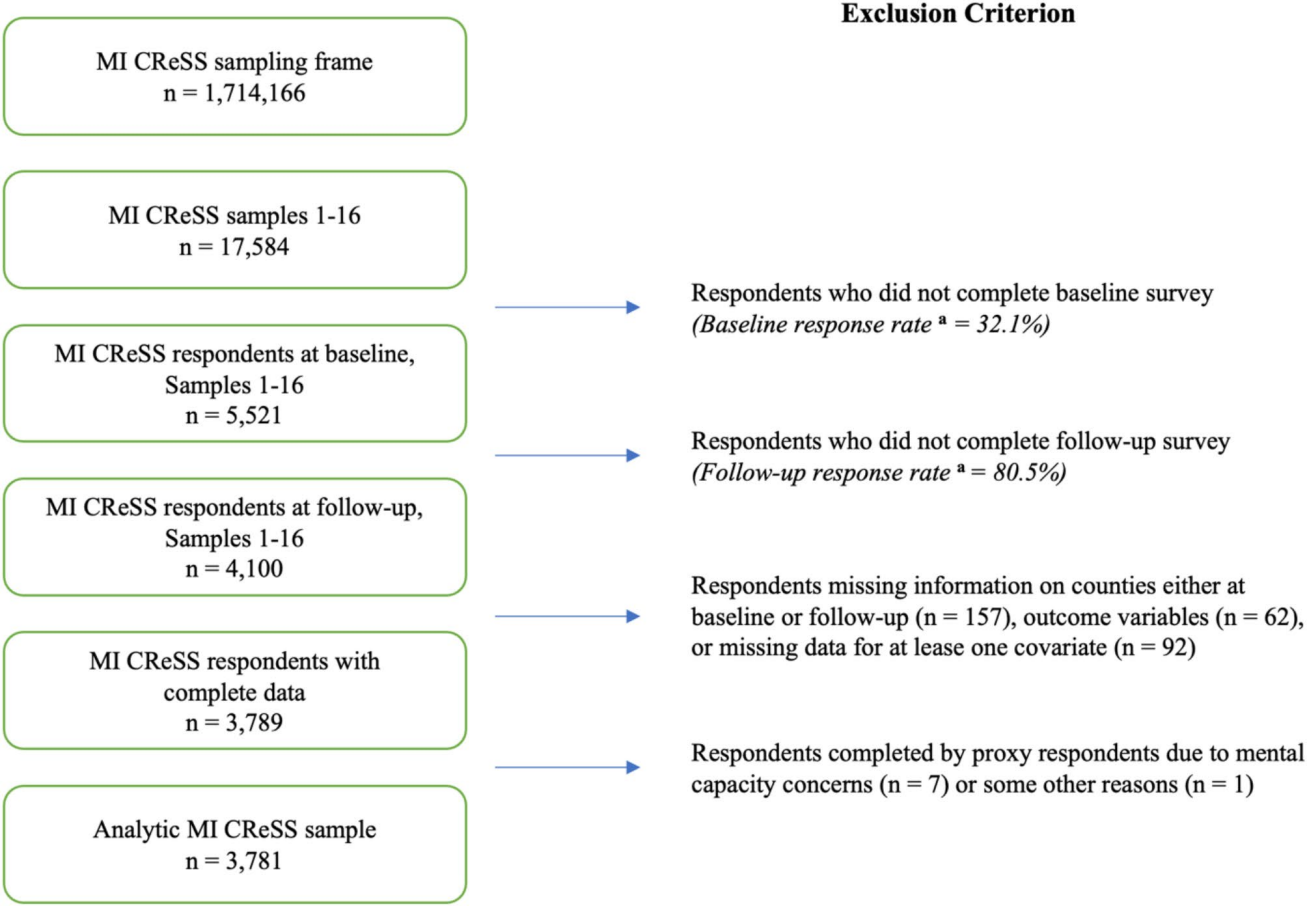
Flowchart for the unweighted analytic sample, Michigan COVID-19 Recovery Surveillance Study, 2020–2023. ^a^ The numerator of the response rate includes both partial and complete surveys

Our analysis included respondents who completed both baseline and follow-up surveys. Of the 4,100 follow-up surveys completed, we excluded surveys missing information about counties (*n* = 157), outcome variables (*n* = 62), or covariates (*n* = 92). We further excluded ten surveys completed by proxy respondents due to mental capacity concerns (*n* = 7) or some other reasons (*n* = 1) at follow-up, leading to an analytic sample of *n* = 3,781 (Fig. 1).

### Outcome variable

We used self-reported measurements of long COVID during the follow-up survey, a median of 18.8 months after respondents’ initial infection using two items: (1) ongoing long COVID and (2) long COVID diagnosis. Prior to asking about long COVID, we provided the definition of long COVID: “The next set of questions ask you about potential long-term symptoms you experienced during your COVID-19 illness and your experiences seeking care for those symptoms. Persistent symptoms of COVID-19 are commonly referred to as Long COVID or Chronic COVID. For this section, we will refer to this condition as Long COVID.” Ongoing long COVID was assessed using two questions. Respondents were asked “At any point since your diagnosis with COVID-19, have you experienced long COVID?” If a respondent answered positively to the question, we also asked, “Have you recovered from long COVID to your usual state of health?” We created a binary variable of ongoing long COVID, which equals 1 if respondents had not recovered from long COVID to their usual state of health, and 0 if respondents had not experienced long COVID or had recovered from long COVD. We assessed a long COVID diagnosis using the question, “Has your doctor or other health professionals told you that you have long COVID, or that you were experiencing long-term symptoms of COVID-19?”

### Social vulnerability data

We obtained county-level publicly available data on the MHSVI in 2020 from the U.S. Department of Health and Human Services Office of Minority Health (OMH) website (https://minorityhealth.hhs.gov/minority-health-svi). The MHSVI was specifically developed for the COVID-19 pandemic to improve existing resources to support the identification of racial and ethnic minoritized communities at highest risk for the disproportionate impact and adverse outcomes due to the pandemic [28]. Therefore, the MHSVI is a more comprehensive index to study COVID-19-related resources and outcomes compared to the standard SVI. Using 5-year estimates of demographic data from the U.S. Census Bureau’s American Community Survey 2016–2020, the MHSVI estimates the relative vulnerability of each county in the U.S. by subsuming 34 social factors in six themes: socioeconomic status, household composition and disability status, racial and ethnic minority status and language, housing type and transportation, healthcare infrastructure and access, and medical vulnerability [28, 31]. For each county, the 34 social factors were ranked and assigned a percentile-rank value ranging from 0 to 1, with higher percentiles indicating higher social vulnerability. Then, the score for each theme was obtained by summing and ranking the percentile values. The scores of six themes were summed and ranked to generate the overall MHSVI ranging from 0 to 1, with a higher score indicating higher social vulnerability. Additional details on MHSVI variable selection and methods are available elsewhere [31]. We merged the MHSVI data with data from the MI CReSS based on addresses recorded for each respondent in the MDSS at time of COVID-19 illness onset. Approximately 92% of respondents lived in the same county at baseline and follow-up, and 4.4% lived in different counties. The remaining 3.6% of respondents had missing information on counties either at baseline or follow-up and were excluded from the analysis. Then, we created a binary variable of the MHSVI using a cut-point of ≥ 75th percentile to indicate high social vulnerability overall and by each theme based on previous literature [24, 32–37]. Additionally, we used quintiles of the MHSVI as a sensitivity analysis (Q1 = lowest [reference], Q5 = highest) [38].

### Covariates

We included individual-level sociodemographic factors at follow-up survey as covariates: age group (18 − 34, 35 − 54, 55 − 64, ≥ 65), sex at birth (male, female), race and ethnicity (Hispanic, non-Hispanic White, non-Hispanic Black, another non-Hispanic race or ethnicity), education (high school or less, some college, college graduate), household income (<$35,000, $35,000 − 74,999, ≥$75,000), and health insurance status (private insurance, Medicare/Medicaid/another type, none). We adjusted for survey-related factors including mode of completion (phone or online) and pandemic phase based on when the respondent was diagnosed with COVID-19 (phase 1: March 2020–September 2020, phase 2: October 2020–February 2021, phase 3: March 2021–September 2021, phase 4: October 2021–May 2022). We also added county-level covariates: population size from the 2020 U.S. decennial Census and rural-urban classification from the 2023 U.S. Department of Agriculture-Economic Research Service [9].

### Statistical analysis

First, we calculated weighted descriptive statistics to characterize the analytic sample. Next, we calculated weighted prevalence estimates of ongoing long COVID and long COVID diagnosis by MHSVI and covariates. Statistical differences were evaluated with Pearson chi-square test with Rao’s correction. Then, we conducted unadjusted and adjusted modified Poisson regression models with robust standard errors to estimate the prevalence ratio (PR) for associations between a county-level binary variable of MHSVI and individual-level ongoing long COVID and long COVID diagnosis. The analyses were completed for overall MHSVI as well as each theme of MHSVI (socioeconomic status, household composition and disability status, racial and ethnic minority status and language, housing type and transportation, healthcare infrastructure and access, and medical vulnerability). For the sensitivity analysis, we conducted the same regression models using quintiles of MHSVI. All statistical analyses were completed using Stata, version 17.0. All estimates were weighted to account for nonresponse at baseline and attrition at follow-up [39]. For household income, missing information was imputed using the weighted sequential hot-deck method under a missing at random assumption [40]. We further estimated robust standard errors by clustering respondents within counties.

## Results

Among our study respondents, 17.4% reported they had ongoing long COVID and 11.2% reported they had received a diagnosis of long COVID (Table 1). About 30.6% of respondents reported living in highly socially vulnerable areas. Respondents were predominately <55 years, female, non-Hispanic White, college graduate, had a household income ≥$75,000, insured, and lived in counties with ≥700,000 population and classified as urban.

**Table 1:** Characteristics of study participants, Michigan COVID-19 recovery surveillance study, 2022–2023 (*n* = 3,781)

	Weighted percentage
Ongoing long COVID	
Yes	17.4
No	82.6
Long COVID diagnosis	
Yes	11.2
No	88.8
Minority Health Social Vulnerability Index	
Low/moderate ^a^	69.4
High ^b^	30.6
Age	
18–34	32.6
35–54	36.0
55–64	16.4
≥65	15.0
Sex	
Male	45.2
Female	54.8
Race and ethnicity	
Hispanic	6.7
NH White	70.6
NH Black	10.4
Another NH race and ethnicity	12.3
Education	
High school or less	23.7
Some college	32.8
College graduate	43.5
Household income	
<$35,000	27.6
$35,000–74,999	29.7
≥$75,000	42.7
Health insurance status	
Private insurance	69.0
Medicare/Medicaid/another type	24.3
None	6.7
County-level population size	
<180,000	30.3
180,000–699,999	29.1
≥700,000	40.7
County-level rural-urban classification	
Urban	86.7
Rural	13.3

COVID-19 = Coronavirus disease 2019, NH = non-Hispanic

a. <75th percentile

b. ≥75th percentile

The prevalence of ongoing long COVID was 17.2% among respondents residing in highly socially vulnerable counties and 17.5% among respondents residing in the low-to-moderately socially vulnerable counties, which was not statistically different (*p* = 0.882) (Table 2). The prevalence of long COVID diagnosis was 12.2% among respondents in highly socially vulnerable counties, higher than the prevalence among respondents in low-to-moderately socially vulnerable counties (10.7%), but the difference was not statistically significant (*p* = 0.264). There were statistical differences in the weighted prevalence of both ongoing long COVID and long COVID diagnosis across age, sex, race and ethnicity, education, household income, and health insurance status categories. The prevalence of both ongoing long COVID and long COVID diagnosis was highest among respondents who were aged 55–64, female, non-Hispanic Black, had some college education, had a household income <$35,000, and had Medicare/Medicaid/another type of health insurance. The prevalence of ongoing long COVID was highest among respondents living in counties with a population of less than 180,000, and the prevalence of long COVID diagnosis was higher among respondents living in rural areas than those living in urban areas.

**Table 2:** Weighted prevalence of ongoing long COVID and long COVID diagnosis by minority health social vulnerability index and covariate among study participants, Michigan COVID-19 recovery surveillance study, 2022–2023 (*n* = 3,781)

	Ongoing long COVID	*p*-value ^a^	Long COVID diagnosis	*p*-value ^a^
Minority Health Social Vulnerability Index		0.882		0.264
Low/moderate ^b^	17.5		10.7	
High ^c^	17.2		12.2	
Age		< 0.001		< 0.001
18–34	10.5		7.5	
35–54	20.5		12.1	
55–64	21.6		15.2	
≥65	20.3		12.6	
Sex		< 0.001		< 0.001
Male	12.7		8.3	
Female	21.2		13.6	
Race and ethnicity		0.005		0.012
Hispanic	16.7		10.0	
NH White	16.6		10.3	
NH Black	24.4		15.8	
Another NH race and ethnicity	16.1		12.9	
Education		< 0.001		0.046
High school or less	18.8		12.4	
Some college	19.8		12.4	
College graduate	14.7		9.5	
Household income		< 0.001		< 0.001
<$35,000	23.2		15.8	
$35,000–74,999	17.5		12.0	
≥$75,000	13.5		7.6	
Health insurance status		0.001		< 0.001
Private insurance	16.0		10.0	
Medicare/Medicaid/another type	21.0		15.1	
None	18.0		8.8	
County-level population size		< 0.001		0.076
<180,000	19.1		12.5	
180,000–699,999	14.0		9.5	
≥700,000	18.4		11.3	
County-level rural-urban classification		0.669		0.017
Urban	17.2		10.6	
Rural	18.1		14.6	

COVID-19 = Coronavirus disease 2019, NH = non-Hispanic

a. Statistical differences were evaluated with Pearson chi-square test with Rao’s correction

b. <75th percentile

c. ≥75th percentile

In unadjusted regression models, living in highly socially vulnerable counties was not associated with the prevalence of ongoing long COVID compared to living in low-to-moderately socially vulnerable counties (PR: 0.98, 95% confidence interval [CI]: 0.78–1.24) (Table 3). There continued to be no association between living in a highly socially vulnerable county and ongoing long COVID after adjustment for individual-level and county-level covariates (aPR: 0.94, 95% CI: 0.79–1.13). Moreover, residing in highly socially vulnerable counties, compared to residing in low-to-moderately socially vulnerable counties, was not associated with a prevalence of long COVID diagnosis in the unadjusted model (PR: 1.14, 95% CI: 0.91–1.43) or the adjusted model (aPR: 1.15, 95% CI: 0.93–1.42).

**Table 3:** Associations of minority health social vulnerability index with ongoing long COVID and long COVID diagnosis, Michigan COVID-19 recovery surveillance study, 2022–2023 (*n* = 3,781)

	Ongoing long COVID		Long COVID diagnosis	
	Unadjusted PR (95% CI)	Adjusted PR (95% CI)	Unadjusted PR (95% CI)	Adjusted PR (95% CI)
Minority Health Social Vulnerability Index (ref: low/moderate ^b^)				
High ^c^	0.98	0.94	1.14	1.15
	(0.78–1.24)	(0.79–1.13)	(0.91–1.43)	(0.93–1.42)
Age (ref: 18–34)				
35–54		2.01***		1.66***
		(1.76–2.29)		(1.36–2.03)
55–64		2.10***		2.17***
		(1.76–2.52)		(1.58–2.98)
≥65		1.76***		1.47*
		(1.46–2.12)		(1.09–1.98)
Sex (ref: male)				
Female		1.63***		1.55***
		(1.45–1.84)		(1.35–1.79)
Race and ethnicity (ref: NH White)				
Hispanic		0.81		0.78
		(0.55–1.19)		(0.54–1.12)
NH Black		1.11		1.14
		(0.89–1.40)		(0.88–1.47)
Another NH race or ethnicity		0.92		1.17
		(0.70–1.20)		(0.92–1.48)
Education (ref: college graduate)				
< High school		0.90		0.81†
		(0.75–1.07)		(0.65–1.02)
Some college		1.08		0.99
		(0.94–1.24)		(0.81–1.21)
Household income (ref: ≥$75,000)				
<$35,000		1.61**		1.94***
		(1.21–2.13)		(1.42–2.66)
$35,000–74,999		1.30**		1.59***
		(1.08–1.58)		(1.29–1.95)
Health insurance status (ref: private insurance)				
Medicare/Medicaid/another type		1.36*		0.97
		(1.05–1.76)		(0.61–1.56)
None		1.07		1.13
		(0.87–1.31)		(0.91–1.41)
County-level population size (ref: ≥700,000)				
<180,000		1.12		1.09
		(0.95–1.32)		(0.85–1.39)
180,000–699,999		0.86*		0.97
		(0.76–0.97)		(0.79–1.19)
County-level rural-urban classification (ref: urban)				
Rural		0.90		1.36*
		(0.70–1.14)		(1.03–1.81)

COVID-19 = coronavirus disease 2019, PR = prevalence ratio, CI = confidence interval, NH = non-Hispanic

Notes: Adjusted model included mode of interview and pandemic phase

b. <75th percentile

c. ≥75th percentile

*** *p* < 0.001, ** *p* < 0.01, * *p* < 0.05, † *p* < 0.1

The associations, however, differed by theme of MHSVI (Table 4). The medical vulnerability theme presented robust results: respondents in highly socially vulnerable counties had 1.44 times higher prevalence of long COVID diagnosis (95% CI: 1.29–1.60) in the unadjusted model, which was slightly attenuated to 1.32 in the adjusted model but remained statistically significant (95% CI: 1.12–1.57). In other themes, there were no statistically significant associations between living in highly socially vulnerable counties and long COVID measurements after the adjustment for individual-level and county-level covariates. In the socioeconomic status theme, respondents residing in highly socially vulnerable counties had 1.15 times higher prevalence of ongoing long COVID (95% CI: 1.00–1.31) and 1.38 times higher prevalence of long COVID diagnosis (95% CI: 1.23–1.56), which were not statistically significant in the adjusted model. Respondents residing in highly socially vulnerable counties for the minority status and language theme had 1.24 times higher prevalence of long COVID diagnosis (95% CI: 1.05–1.47), but it was not statistically significant after the adjustment for covariates.

**Table 4:** Associations of each theme of minority health social vulnerability index with ongoing long COVID and long COVID diagnosis, Michigan COVID-19 recovery surveillance study, 2022–2023 (*n* = 3,781)

	Ongoing long COVID		Long COVID diagnosis	
	Unadjusted PR (95% CI)	Adjusted PR (95% CI)	Unadjusted PR (95% CI)	Adjusted PR (95% CI)
**Theme of Minority Health Social Vulnerability Index**				
Socioeconomic status (ref: low/moderate ^c^)				
High ^c^	1.15*	0.99	1.38***	1.18
	(1.00–1.31)	(0.84–1.16)	(1.23–1.56)	(0.97–1.45)
Household composition and disability (ref: low/moderate ^c^)				
High ^c^	1.08	1.04	1.20	1.22†
	(0.87–1.35)	(0.91–1.19)	(0.94–1.52)	(0.99–1.50)
Minority status and language (ref: low/moderate ^c^)				
High ^c^	1.08	0.94	1.24*	1.16
	(0.88–1.32)	(0.76–1.17)	(1.05–1.47)	(0.91–1.48)
Housing type and transportation (ref: low/moderate ^c^)				
High ^c^	0.83†	0.86†	0.92	0.97
	(0.67–1.02)	(0.74–1.01)	(0.74–1.15)	(0.79–1.19)
Healthcare infrastructure and access (ref: low/moderate ^c^)				
High ^c^	0.92	0.93	0.87	0.82†
	(0.75–1.13)	(0.79–1.10)	(0.68–1.11)	(0.65–1.02)
Medical vulnerability (ref: low/moderate ^c^)				
High ^c^	1.22***	1.09	1.44***	1.32**
	(1.11–1.34)	(0.98–1.20)	(1.29–1.60)	(1.12–1.57)

COVID-19 = coronavirus disease 2019, PR = prevalence ratio

Notes: Adjusted model included age, sex, race and ethnicity, education, household income, health insurance status, population size, rural-urban classification, mode of interview, and pandemic phase

b. <75th percentile

c. ≥75th percentile

*** *p* < 0.001, ** *p* < 0.01, * *p* < 0.05, † *p* < 0.1

The sensitivity analysis indicated that the overall results were similar, but statistical significance was more pronounced when we used quintiles of the MHSVI (Supplementary Table S1). Respondents living in the highest socially vulnerable counties (Q5) had 1.48 times higher prevalence of long COVID diagnosis (95% CI: 1.01–2.17) than those living in the lowest socially vulnerable counties (Q1) with adjustment for covariates. By theme of the MHSVI, respondents in the more socially vulnerable counties had a higher prevalence of ongoing long COVID (aPR: 1.14, 95% CI: 1.02–1.27 for Q3) and long COVID diagnosis (aPR: 1.26, 95% CI: 1.01–1.56 for Q3; aPR: 1.41, 95% CI: 1.10–1.79 for Q4; aPR: 1.43, 95% CI: 1.16–1.76 for Q5) in the socioeconomic status theme. In the household composition and disability theme, respondents in the more socially vulnerable counties had a higher prevalence of ongoing long COVID (aPR: 1.24, 95% CI: 1.00–1.54 for Q2) and long COVID diagnosis (aPR: 1.41, 95% CI: 1.06–1.86 for Q2; aPR: 1.35, 95% CI: 1.07–1.71 for Q3; aPR: 1.64, 95% CI: 1.17–2.30 for Q5). The trend was similar in in the housing and transportation theme for ongoing long COVID (aPR: 1.19, 95% CI: 1.05–1.35 for Q2) and long COVID diagnosis (aPR: 1.32, 95% CI: 1.11–1.57 for Q2; aPR: 1.40, 95% CI: 1.12–1.76 for Q3; aPR: 1.36, 95% CI: 1.06–1.75 for Q4). Our results for the medical vulnerability theme were robust, suggesting that respondents in highly socially vulnerable counties had a higher prevalence of long COVID diagnosis compared to those in the lowest socially vulnerable counties (aPR: 1.18, 95% CI: 1.00–1.38 for Q3; aPR: 1.36, 95% CI: 1.10–1.68 for Q4). In contrast, living in highly socially vulnerable counties in terms of healthcare infrastructure and access was associated with a lower prevalence of long COVID diagnosis (aPR: 0.76, 95% CI: 0.57–1.00 for Q3; aPR: 0.71, 95% CI: 0.54–0.93 for Q5).

## Discussion

This study examines how social vulnerability is related to long COVID illness by using population-based data of adults with COVID-19 infection. With the overall MHSVI score, living in highly socially vulnerable counties was not associated with the prevalence of ongoing long COVID or long COVID diagnosis compared to living in low-to-moderately socially vulnerable counties. However, the theme analysis indicated that living in counties with high medical vulnerability (i.e., higher rates of cardiovascular disease, chronic respiratory disease, obesity, diabetes, no internet access) was associated with a higher prevalence of long COVID diagnosis, which was robust in both main and sensitivity analysis. Our findings highlight the importance of studying how different aspects of area-level social vulnerability contribute to health inequity.

Among our analytic sample of adults with COVID-19 in Michigan, 17.4% reported ongoing long COVID and 11.2% received a diagnosis of long COVID. As previous studies used different study populations, methods, and definitions of long COVID, it is difficult to compare the prevalence of long COVID across studies. One comparable estimate was from the Household Pulse Survey, which suggested that roughly 10% of U.S. adults with COVID-19 reported ongoing long COVID as of October 2023 [41]. The prevalence of ongoing long COVID was higher in our study, possibly because we included only PCR-confirmed COVID-19 cases and defined long COVID as potential long-term symptoms without a specific time frame, whereas the Household Pulse Survey identified COVID-19 cases based on any type of COVID-19 tests and defined long COVID as long-term symptoms lasting 3 months or longer. For example, individuals with severe symptoms of COVID-19 illness, who are more likely to experience long COVID [18], may also be more likely to seek medical care and get PCR-confirmed tests.

The main finding on the association between overall MHSVI and long COVID was not statistically significant, while the sensitivity analysis indicated that only respondents in the highest MHSVI quintile had a higher prevalence of long COVID diagnosis than those in the lowest MHSVI quintile. However, previous studies found statistically significant associations of higher MHSVI with COVID-19 incidence [2], mobility disability after a COVID-19 diagnosis [24], and COVID-19 mortality [2]. Additionally, a few studies have found areas with vulnerable socioeconomic status (e.g., households in poverty) or household composition and disability (e.g., households with children or older adults) are more likely to have worse COVID-19-related outcomes, such as COVID-19 incidence [9] or mobility disability after a COVID-19 diagnosis [24]. Our findings on the robust relationship between higher medical vulnerability and long COVID diagnosis are aligned with a recent study, which suggested that counties with greater medical vulnerabilities had lower COVID-19 vaccination coverage [42] given that COVID-19 vaccines protect against long COVID [20]. This could be because the risk of having long COVID illness is better explained by medical vulnerability than other aspects of social vulnerability when individual-level and area-level covariates were adjusted. Our findings may reflect that communities with vulnerable medical conditions (e.g., cardiovascular or chronic respiratory disease) have (1) lower capacities and fewer resources to recover from disasters such as COVID-19 pandemic [43, 44], (2) less access to healthcare services with high quality to receive treatment [9], and (3) a higher burden of other chronic conditions and comorbidities that worsen long-term effects of COVID illness [45, 46]. While the sensitivity analysis for most themes indicated a positive association between social vulnerability and long COVID, living in socially vulnerable counties assessed by the healthcare infrastructure and access theme was associated with a lower prevalence of long COVID diagnosis. This might be because individuals in these areas have limited access to medical services needed to receive a long COVID diagnosis, resulting in under-reporting. It has been documented that restricted healthcare access can lead to underestimating cases of infectious diseases [47]. Further research should be done to identify the mechanism underlying the observed association.

Our study has several limitations. First, our data included only adults who had a positive PCR test for SARS-CoV-2, were recorded in the MDSS with valid contact and geographic information,

were alive when the survey sample was drawn, and agreed to participate at follow-up. Thus, our sample may have selection bias as we did not include individuals who had COVID-19 but were never tested or those who tested positive at home and individuals who died from severe COVID-19 illness, which limits generalizability. Second, we used self-reported assessments of ongoing long COVID and long COVID diagnosis by health professionals, which lack medical confirmation. As the long COVID definitions have been evolving due to highly heterogeneous and complex symptoms of long COVID [48, 49], our long COVID measurements might not correctly assess ongoing long COVID or long COVID diagnosis. Additionally, responses to our long COVID questions could be different due to differences between individuals, such as health knowledge, health-seeking behaviors, access to healthcare, and availability of clinicians [50, 51]. Third, the response rate for the baseline survey was 32.1%, which is consistent with other large probability surveys [52, 53]. To account for nonresponse, we used sampling weights that matched the age and sex distribution of the sampling frame. Additionally, a nonresponse bias analysis demonstrated few differences between respondents and nonrespondents [54]. Finally, causal inference is limited because this study examined cross-sectional associations between social vulnerability and long COVID using observational data.

Despite these limitations, our study contributes to the literature by identifying understudied associations between area-level social vulnerability and long COVID, using a population-based study, which provides timely and representative data during the public health emergency. Using the MHSVI, a specific and comprehensive social vulnerability index to study COVID-19 pandemic, we found that living in medically vulnerable areas was associated with long COVID diagnosis. Community-based support is needed for vulnerable areas with high concentrations of households with pre-existing chronic conditions. Support could include improving access and quality of medical care services and healthcare providers for long COVID illness for households with chronic conditions [43]. Additionally, social support programs collaborating with social workers (e.g., group wellness program, care coordination) may be helpful to reduce stress, facilitate healthcare utilization, and ease burdens of comorbidities [55]. As existing inequalities are magnified during times of crisis, governments and public health authorities should pay close attention to upstream social determinants of health and provide medically vulnerable communities with more resources particularly during pandemics or other public health emergencies.

## Electronic supplementary material

Below is the link to the electronic supplementary material.

Supplementary Material 1

## Data Availability

No datasets were generated or analysed during the current study.
