# Supplementary material for: Minority health social vulnerability index and long COVID illness among a statewide, population-based study of adults with polymerase chain reaction-confirmed SARS-CoV-2
Source: Arch Public Health. 2025 Mar 10;83:64. doi: 10.1186/s13690-025-01553-z (PMC11892128; doi:10.1186/s13690-025-01553-z)
Supplement: Supplementary file 1 — Supplementary Material 1 [file 13690_2025_1553_MOESM1_ESM.docx]

**Supplementary Table 1.** Associations of quintiles of Minority Health Social Vulnerability Index with ongoing long COVID and long COVID diagnosis, Michigan COVID-19 Recovery Surveillance Study, 2022–2023 (n = 3,781)

|  | **Ongoing long COVID** | | **Long COVID diagnosis** | |
| --- | --- | --- | --- | --- |
|  | Unadjusted  PR (95% CI) | Adjusted  PR (95% CI) | Unadjusted  PR (95% CI) | Adjusted  PR (95% CI) |
| Minority Health Social Vulnerability Index (ref: Q1, lowest) |  |  |  |  |
| Q2 | 1.18† | 1.17 | 1.00 | 1.17 |
|  | (0.97–1.44) | (0.93–1.47) | (0.79–1.27) | (0.83–1.64) |
| Q3 | 0.97 | 1.06 | 1.03 | 1.17 |
|  | (0.79–1.19) | (0.86–1.30) | (0.74–1.44) | (0.87–1.58) |
| Q4 | 1.05 | 1.11 | 1.09 | 1.30† |
|  | (0.85–1.30) | (0.90–1.35) | (0.87–1.37) | (0.97–1.76) |
| Q5 (highest) | 1.20 | 1.11 | 1.33* | 1.48* |
|  | (0.94–1.51) | (0.83–1.48) | (1.05–1.69) | (1.01–2.17) |
| **By theme** |  |  |  |  |
| Socioeconomic status (ref: Q1, lowest) |  |  |  |  |
| Q2 | 0.97 | 1.10 | 1.10 | 1.16 |
|  | (0.87–1.10) | (0.95–1.27) | (0.93–1.29) | (0.93–1.43) |
| Q3 | 1.20** | 1.14* | 1.39** | 1.26* |
|  | (1.06–1.37) | (1.02–1.27) | (1.12–1.71) | (1.01–1.56) |
| Q4 | 1.31* | 1.19† | 1.66*** | 1.41** |
|  | (1.05–1.64) | (0.97–1.47) | (1.36–2.01) | (1.10–1.79) |
| Q5 (highest) | 1.30*** | 1.11 | 1.72*** | 1.43*** |
|  | (1.13–1.49) | (0.94–1.31) | (1.48–2.00) | (1.16–1.76) |
|  |  |  |  |  |
| Household composition, disability (ref: Q1, lowest) |  |  |  |  |
| Q2 | 1.17 | 1.24* | 1.37* | 1.41* |
|  | (0.92–1.50) | (1.00–1.54) | (1.02–1.84) | (1.06–1.86) |
| Q3 | 1.12 | 1.19† | 1.14 | 1.35* |
|  | (0.92–1.36) | (0.99–1.43) | (0.97–1.35) | (1.07–1.71) |
| Q4 | 0.87 | 1.00 | 0.84† | 1.11 |
|  | (0.71–1.06) | (0.81–1.23) | (0.70–1.02) | (0.84–1.47) |
| Q5 (highest) | 1.25* | 1.18 | 1.42*** | 1.64** |
|  | (1.04–1.52) | (0.92–1.52) | (1.21–1.68) | (1.17–2.30) |
| Minority status and language (ref: Q1, lowest) |  |  |  |  |
| Q2 | 0.96 | 0.96 | 0.90 | 0.96 |
|  | (0.75–1.22) | (0.79–1.17) | (0.66–1.23) | (0.75–1.24) |
| Q3 | 0.90 | 0.96 | 0.73* | 0.87 |
|  | (0.75–1.09) | (0.76–1.20) | (0.55–0.98) | (0.63–1.21) |
| Q4 | 0.90 | 0.96 | 0.85 | 1.02 |
|  | (0.72–1.12) | (0.77–1.19) | (0.67–1.07) | (0.75–1.39) |
| Q5 (highest) | 1.01 | 0.91 | 1.06 | 1.09 |
|  | (0.78–1.31) | (0.66–1.23) | (0.82–1.38) | (0.77–1.55) |
| Housing and transportation (ref: Q1, lowest) |  |  |  |  |
| Q2 | 1.29*** | 1.19** | 1.48*** | 1.32** |
|  | (1.13–1.46) | (1.05–1.35) | (1.28–1.71) | (1.11–1.57) |
| Q3 | 1.23** | 1.12 | 1.63*** | 1.40** |
|  | (1.07–1.41) | (0.96–1.31) | (1.34–1.96) | (1.12–1.76) |
| Q4 | 0.94 | 1.02 | 1.25* | 1.36* |
|  | (0.80–1.10) | (0.87–1.19) | (1.05–1.48) | (1.06–1.75) |
| Q5 (highest) | 0.92 | 0.93 | 1.25† | 1.26† |
|  | (0.74–1.15) | (0.77–1.12) | (1.00–1.57) | (0.96–1.66) |
| Healthcare infrastructure and access (ref: Q1, lowest) |  |  |  |  |
| Q2 | 0.95 | 1.00 | 1.04 | 1.03 |
|  | (0.81–1.10) | (0.89–1.13) | (0.86–1.26) | (0.84–1.25) |
| Q3 | 0.93 | 0.93 | 0.84 | 0.76* |
|  | (0.76–1.14) | (0.72–1.19) | (0.67–1.06) | (0.57–1.00) |
| Q4 | 0.83* | 0.91 | 0.88 | 0.83 |
|  | (0.71–0.96) | (0.77–1.09) | (0.68–1.13) | (0.66–1.04) |
| Q5 (highest) | 0.92 | 0.90 | 0.86 | 0.71* |
|  | (0.75–1.13) | (0.72–1.12) | (0.63–1.18) | (0.54–0.93) |
| Medical vulnerability (ref: Q1, lowest) |  |  |  |  |
| Q2 | 0.89* | 0.89 | 1.02 | 0.95 |
|  | (0.81–0.98) | (0.76–1.03) | (0.83–1.26) | (0.74–1.21) |
| Q3 | 1.09 | 0.98 | 1.32*** | 1.18* |
|  | (0.92–1.28) | (0.87–1.10) | (1.13–1.55) | (1.00–1.38) |
| Q4 | 1.30*** | 1.05 | 1.61*** | 1.36** |
|  | (1.16–1.47) | (0.92–1.20) | (1.38–1.89) | (1.10–1.68) |
| Q5 (highest) | 1.24* | 1.06 | 1.53*** | 1.21 |
|  | (1.04–1.47) | (0.93–1.21) | (1.28–1.83) | (0.94–1.56) |

COVID-19 = coronavirus disease 2019, PR = prevalence ratio, CI = confidence interval, NH = non-Hispanic

Notes: Adjusted model included age, sex, race and ethnicity, education, household income, health insurance status, population size, rural-urban classification, mode of interview, and pandemic phase.

*** p < 0.001, ** p < 0.01, * p < 0.05, † p < 0.1
